# Atypical presentation of "takotsubo cardiomyopathy" without ST segment elevation: a case report

**DOI:** 10.1186/1757-1626-1-309

**Published:** 2008-11-14

**Authors:** Yen-Wen Liu, Ju-Yi Chen, Wei-Chuan Tsai, Jyh-Hong Chen

**Affiliations:** 1 Department of Internal Medicine, National Cheng Kung University Medical Center, Tainan, Taiwan; 2 Division of Cardiology, Department of Internal Medicine, National Cheng Kung University Hospital Dou-Liou Branch, Dou-Liou, Taiwan

## Abstract

**Introduction:**

"Takotsubo cardiomyopathy" is characterized by transient LV dysfunction and mimicking acute myocardial infarction.

**Case presentation:**

We reported a case with atypical presentation of "takotsubo cardiomyopathy" without ST segment elevation, but with severe transient left ventricular dysfunction.

**Conclusion:**

Diagnosis of "takotsubo cardiomyopathy" should be based on typical left ventricular dysfunction without coronary artery disease.

## Introduction

"Takotsubo cardiomyopathy", firstly described by Satoh et al. and Dote et al. in 1990, is named due to reversible abnormal wall motion of the left ventricle (LV) with a systolic shape on left ventriculography similar to the shape of a Japanese fishing pot for octopus, a unique shape with a round bottom and narrow neck.[1] The wall motion abnormalities in this disease exhibit both apical akinesis and basal hyperkinesis in the acute phase. Yet the abnormality is not related to the coronary blood distribution. The LV dysfunction will be normalized within a few weeks, and without any sequalae.[2] This is quite different from the wall motion abnormalities seen in acute myocardial infarction or acute myocarditis. Besides, diffuse T wave inversion, a prolonged QT interval, and greater ST-segment elevation in leads V4–6 without reciprocal changes occurred in most patients.[3,4]

Notably, the onset is usually preceded by emotional stress or prior aggravation of an underlying disorder (for example, cerebrovascular accident, epilepsy, or acute abdomen etc.).[5] It suggests that emotional or physical stress may play an important role in takostubo cardiomyopathy. In the case we reported, the patient exhibited a typical LV abnormality seen in takotsubo cardiomyopathy, but without any preceding emotional or physical stress and typical electrocardiogram (ECG) change.

## Case Report

**Figure 1:**
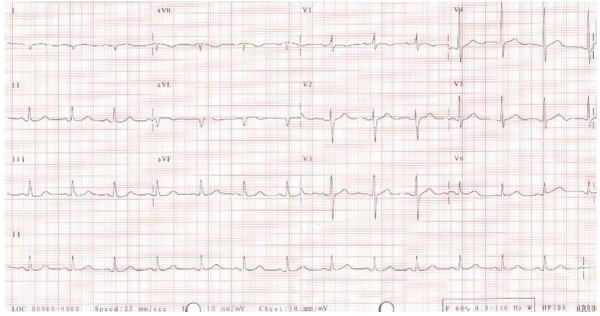
Initial electrocardiography (ECG) during attack.

**Figure 2:**
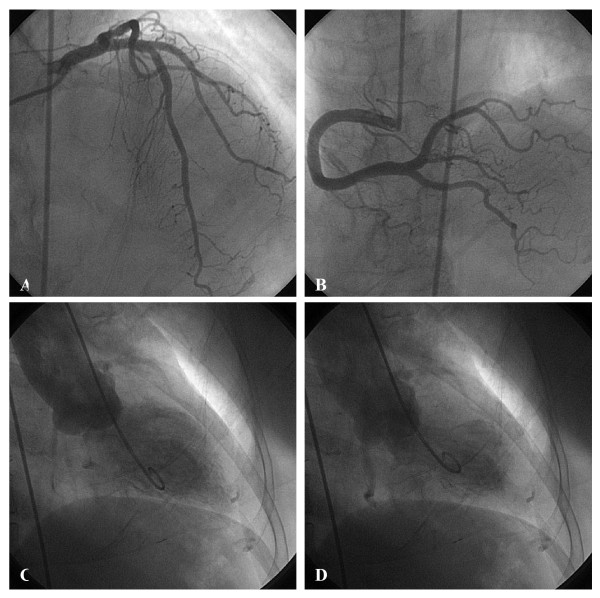
No significant stenosis is seen on coronary angiogram: (A) left coronary artery (left anterior oblique view); (B) right coronary artery (right anterior oblique view); left ventriculography (right anterior oblique view): (C) end-diastole; (D) end-systole shows apical ballooning with basal segments hyperkinesis.

A 65-year-old female was relatively well before and had history of diabetes mellitus without medical control. This admission, she suffered from sudden onset of retrosternal chest pain at rest and came to our emergency room for help. The pain was tight in characteristic and radiation to the epigastric area. On physical examination, she had a blood pressure of 125/88 mmHg, heart rate of 77/minutes, and clear consciousness. Crackle could be heard in both lung fields and grade three holosystolic murmur was heard in the left lower sternal border. The blood tests revealed creatinine of 0.9 mg/dl, creatinine kinase of 251 IU/L, creatinine kinase MB of 31.11 ng/ml, and Troponin T of 0.800. The peak of serial cardiac enzymes values were as followed: creatinine kinase 500, creatinine kinase MB 40 and Troponin T 1.09 which noted 24 hours after onset. Chest X-ray showed mild pulmonary congestion. An ECG showed rightward axis, normal QT interval with QTc of 422 ms, and non-significant ST-T elevation (0.05 mv) at leads I, aVL, and V5 (Fig. 1) without dynamic change in the serial follow-up ECGs. Under the impression of non-ST elevation myocardial infarction, conservative treatment with anti-thrombotic and anti-platelet regimens was prescribed. The echocardiography showed impaired global LV systolic performance with mid-septum hypokinesis and akinesis of mid-anterior to apical-anterior wall, but well preserved basal septal contractility. Cardiac catheterization was performed on the 7^th ^day. Coronary angiogram revealed no significant stenonsis on coronary arteries and left ventriculography showed anterior wall akinesis, apical hypokinesis and slight basal hyperkinesis of left ventricle (Fig. 2). The patient was improved after supportive care and follow-up echocardiography showed complete recovery of left ventricular systolic function without abnormal regional wall motion 2 weeks later.

## Discussion

There are several reports of patients with profound, reversible LV dysfunction, especially after sudden emotional stress. [5-7] This pattern of LV dysfunction has been referred to as "takotsubo cardiomyopathy". It is characterized by transient LV dysfunction with chest pain, electrocardiographic changes, especially ST segment elevation, and minimal release of myocardial enzymes mimicking acute myocardial infarction. Left ventriculograms showed apical ballooning with basal hyperkinesias.[4,7] Diagnosis of "takotsubo cardiomyopathy" is based on the following characteristics: 1) onset symptoms resembling those of acute myocardial infarction, 2) apical akinesis and basal hyperkinesis, 3) ST segment elevation with T wave inversion and QT prolongation, 4) minimal myocardial enzymes release, 5) no angiographical stenosis in the coronary arteries, and 6) reversible left ventricular function.[1,8] The following features of the patient we reported are consistent with typical "takotsubo cardiomyopathy": 1) minimal myocardial enzymes release; 2) an absence of organic coronary artery lesions; 3) a typical "round bottom and narrow neck" shape shown in left ventriculogram; and 4) reversible LV dysfunction, which was recovered in a few weeks and demonstrated by the follow-up echocardiography.

There were features different from the typical presentation of takotsubo cardiomyopathy. First, the degree of ST elevation was not eminent (0.05 mv) and not in the standard leads — the precordial leads, especially V4–6.[4] Tsuchihashi et al.[7] reported that ST-segment elevation was observed in 90% of patients with takotsubo cardiomyopathy. The higher ratio of ST-segment elevation in leads V4–6/V1–3 with the absence of reciprocal changes, and the prolonged QTc interval show a high sensitivity and specificity for diagnosing takotsubo cardiomyopathy.[4]Second, we could not recognize any triggering conditions; the patients denied any emotional or physical stress and there was no abnormal physical examination in the first contact of the patient in the emergent department. However, most of the patients (around 75%) have the triggering conditions, including exposure to internal (emotional) and external stress (physical, exacerbated disorders, procedural and perioperative).[7] As a result, although the patient we reported did not have the typical ECG ST-segment change and the precipitating factor, but had transient LV dysfunction with typical round bottom and narrow neck shape shown in left ventriculogram and normal coronary angiogram, yet takotsubo cardiomyopathy with a rare and atypical presentation was diagnosed.

The precise pathophysiology is still not identified. The mechanism has been reported as stunned myocardium, a prolonged postischemic left ventricular dysfunction after brief myocardial ischemia[9], coronary vasospasm[1], microvascular disturbance in the myocardium[10], acute myocarditis[11] and so on. But in our case, we could not clarify the etiology of transient LV dysfunction. Although the pathophysiology is not clear, yet the mortality of takotsubo cardiomyopathy is very low and the recurrence is rare.[7] However, Sharkey et al. once reported that in acute stage, almost 40% of the patients required aggressive treatment, including hemodynamic stabilization with vasopressor agents and intra-aortic balloon counterpulsation.[12] Unfortunately, there is no independent predictor of deteriorated cardiac failure. Hence, prompt and aggressive pharmacological and hemodynamic support in hemodynamic unstable patients play an important role in the reversal of LV function and survival without sequalae.

## Conclusion

We reported an atypical case of takotsubo cardiomyopathy without typical ECG ST-segment elevation and triggering events. Our case indicated that diagnosis of "takotsubo cardiomyopathy" should be based on typical left ventricular dysfunction without coronary artery disease. The etiology of "takotsubo cardiomyopathy" is probably not solely due to acute stress. Other pathophysiology should be considered.

## Abbreviations

LV: Left ventricle; ECG: electrocardiogram

## Competing interests

The authors declare that they have no competing interests.

## Authors' contributions

YWL wrote the case report, performed the literature review, and obtained the written consent. JYC performed the coronary angiography and left ventriculography, and conceived the study. WCT did literature search and assisted with writing the manuscript. JHC helped to draft the manuscript. All authors have read and approved the final manuscript.

## Consent

Written informed consent was obtained from the publication of this case report and accompanying images. A copy of the written consent is available for review by the Editor-in-Chief of this journal.
